# Burden of chronic disease comorbidities among cancer patients at Queen Elizabeth and Kamuzu Central Hospitals in Malawi: an exploratory cross-sectional study

**DOI:** 10.11604/pamj.2021.40.167.31069

**Published:** 2021-11-17

**Authors:** Jonathan Chiwanda Banda, Adamson Sinjani Muula

**Affiliations:** 1 Department of Public Health, College of Medicine, University of Malawi, Blantyre, Malawi,; 2 Clinical Services Department, Non-Communicable Disease Unit, Ministry of Health, Blantyre, Malawi,; 3 The Africa Center of Excellence in Public Health and Herbal Medicine, College of Medicine, University of Malawi, Blantyre, Malawi

**Keywords:** Chronic disease, comorbidity, cancer patients, Malawi

## Abstract

**Introduction:**

chronic disease comorbidities are common among cancer patients in most parts of the world, however; there are limited data on the same for Malawi. Comorbidities worsen clinical outcomes and are associated with lower quality of life among cancer patients. We aimed at estimating chronic disease comorbidities and associated factors among cancer patients attending oncology services at the Queen Elizabeth Hospital (QECH) and Kamuzu Central Hospital (KCH) in Blantyre and Lilongwe respectively.

**Methods:**

we conducted a cross-sectional study at QECH and KCH in Malawi from January to March 2021. Participants were recruited using simple random sampling technique at the oncology clinics and were interviewed using structured questionnaires. The College of Medicine Research and Ethics Committee (COMREC) approved the study and informed consent was obtained with each participant. Data were analyzed in Stata version 14 and summary statistics were presented as frequencies and proportions.

**Results:**

we interviewed 398 cancer patients and the mean age was 45.4years (SD± 12.77). The common cancers were cervical (30%), Kaposi´s sarcoma (24%), breast (11%), esophageal (4%) and leukemia (4%). The prevalence of chronic disease comorbidities was 61.56% (n=398) and common conditions included: HIV and AIDS (43%), depression (9%) hypertension (8%) and anemia (9%). Chronic disease comorbidities were significantly associated with formal employment (p<0.01) and obesity (p<0.02).

**Conclusion:**

chronic disease comorbidities were prevalent among cancer patients in the study settings in Malawi. There is a need to develop a multidisciplinary approach when managing cancer patients with emphasis on active screening for the common conditions as reported by this study.

## Introduction

Cancer incidence and mortality are rapidly growing worldwide [1]. The GLOBOCAN 2020 report had shown that an estimated 19.3 million new cancer cases and 10.0 million cancer deaths occurred in 2020 [2]. In addition, over 36 million people were living with various forms of cancer and the burden disproportionately affects Low- and Middle-Income Countries (LMICs) which contributed70% of cancer deaths in 2020 [3]. In Malawi, cancers contributed to 16% of Disability-Adjusted Life Years (DALYs) due to Non-Communicable Diseases (NCDs) in 2015 [4]. Cancer survival in Malawi is poor with median survival time of about 9 months and only 6% of patients surviving for 5 years or more [5]. The top five common cancers include: Kaposi sarcoma (34.1%), cancer of the uterine cervix (25.4%), oesophageal (12.0%), non-Hodgkin´s lymphoma (5.7%) and urinary bladder (2.9%) [6].

Many cancer patients also suffer from other chronic comorbidities [7]. These are conditions broadly lasting for longer duration usually one year or more and require ongoing medical attention or limit activities of daily living or both [8]. They may occur prior to or at the same time as the primary disease or afterwards [9]. Several studies across the globe confirm that comorbid chronic conditions are common among cancer patients [7,9-11]. In Malawi, there is data paucity on the comorbid conditions among cancer patients however, in general population, the following estimates were reported: hypertension (33%), anxiety/depression disorders (10-20% )[4], cardiovascular diseases (including vascular disease and stroke), 8.9% [12] while obesity and asthma were estimated at 5%, diabetes mellitus, 6% respectively [13]. HIV prevalence was9.2% in adult population above the age of 18 years [4,14]. Most of these chronic conditions share common risk factors with various forms of cancers and as such, positive associations have been reported among them in several studies and these included the following: ageing above 65 years, physical inactivity, alcohol and smoking histories, obesity and urbanization [1, 7,9-11,13,15-17]. Furthermore, comorbid conditions of cancer patients are significantly associated with worse health status during treatment and oncology follow-up periods as well as low or intermediate socioeconomic status, and poor nutritional status [7,9,10].

Although there is a growing body of research in the field of chronic disease comorbidities among cancer patients in other parts of the world, results from Malawi are almost non-existent [6]. The importance of chronic disease comorbidities among cancer patients cannot be underestimated in relation to cancer diagnosis, management and overall outcomes among patients [5,9]. Therefore, this exploratory study was aimed at estimating pattern and distribution of chronic disease comorbidities among cancer patients attending oncology services at Queen Elizabeth (QECH) and Kamuzu Central Hospitals (KCH) and whether they differed from prevalence estimates in the general population of Malawi. The findings may contribute towards designing appropriate interventions and/or the provision of quality healthcare services and resources for ongoing active surveillance of such conditions among people living with, through and beyond cancer. We hypothesized that prevalence of chronic disease comorbidities among cancer patients in Malawi was greater than 26% due to declining socioeconomic profile which elsewhere was associated with increased proportions of chronic diseases among cancer population [9].

## Methods

**Study setting and design:** we conducted a cross-sectional study at two main referral hospitals of QECH and KCH in Malawi from January 13^th^ and March 23^rd^, 2021. These two facilities attend to most of cancer patients in Malawi. The study took place at oncology clinics which are specialized clinics where all newly diagnosed cancers are referred for further management.

**Sampling size and sampling technique**: sample size was estimated basing on the Cochran´s formula using estimated prevalence of comorbidities at 26% based on recent study in Lagos among cancer patients,5% precision level and 95% confidence level (Z= 1.96) [9]. The calculated sample size was 295 however 398 participants were recruited. We used a simple random sampling using consecutive numbers sampling approach in recruiting participants aged above 18 years of age as they attend adult oncology clinics.

**Inclusion criteria**: all cancer patients above 18 years of age attending to oncology services during the conduct of the study.

**Data collection and data management**: data were collected through face-to-face interviews using a semi-structured questionnaire that was developed by the researcher and was adapted from STEPS survey data tool [18]. The tool was translated into Chichewa language and content validity was assessed by the attending oncologists. We piloted the questionnaire to four participants at each site to estimate average time taken to complete single interview as well as ascertaining clarity of the questions in terms of whether they capture the intended information. The collected data was uploaded into Open Data Kit (ODK) on android tablets to minimize data collection errors and also reduce missing data. Data validations and checks were programmed to ensure that the majority of data capture errors are solved at the data collection point. All data collected on the tablets were being sent to a secure server and routine data quality checks were ran on the server identify any data inconsistencies and discrepancies which were then sent to the data collection teams for resolutions which later applied to the server. Data were downloaded from the server as a CSV dataset which was imported into Stata for further data preparations and data analysis.

**Outcome variable**: the study considered having a chronic disease comorbidity as an outcome. The chronic disease conditions were classified using single count approach and covered most common types of long-term health conditions as adapted from Charlson´s comorbidity index [19,20] and were common in Malawi. The count of chronic health conditions was measured for each respondent based on the number of disease exposures and who had been prescribed medication for their illness. If the respondents had multiple chronic conditions, it was counted as multiple responses. These comorbidities were captured from patients´ files, health passports and self-reports. The conditions included the following: HIV and AIDS, hypertension, diabetes arthritis or osteoporosis, heart disease, diabetes, hypertension, depression, dementia, asthma, or circulatory conditions, malnutrition, myocardial infraction, heart failure, anemia and peptic ulcer disease.

**Independent variables**: the study had the following explanatory variables: (1) sociodemographic characteristics such as sex, age, marital status, area of residence, education level, occupation, socioeconomic status (2); behavioral risk factors such as smoking, alcohol and physical activity (3); cancer diagnosis as it appears in the patient files and the date of diagnosis;(4) cancer stage;(5) intent to treat; (6) and treatment options.

**Data analysis**: we used stata statistical software version 14 Texas 77845 for analysis. Socioeconomic status was generated as a single explanatory variable using factor analysis of five different variables namely: type of residence, house ownership, energy source; water source and type of toilet (flush toilet) because they were all indicators of socioeconomic profile and had ordinal entries. In factor analysis, first level explained largest proportion of total variance and assets that were more unequally distributed across the sample had higher weights. Those weights were used for each asset to generate factor scores. The higher the score indicated the higher the wealth status and vice versa. Finally based on quintiles, the scores were converted into five ordered categories from highest (1^st^ quintile) to lowest (5^th^ quintile). Therefore, the new variable SES was categorized into those five categories namely, highest, higher, high, middle and low. Correlational analyses were done to compare patients with cancer and chronic medical conditions across explanatory variables. Chi-square test was used to find the association between outcome and explanatory variables. We fitted an unadjusted logistic regression model to find the association between outcome and explanatory variables at 5% level. All significant explanatory variables were all fitted into multivariate logistic regression model using forward selection to determine factors significantly associated with chronic disease comorbidity at p<0.05. Sex and age were included in the final model although they were not significant on the unadjusted model because they are known confounders in developing chronic comorbidity among cancer patients. The model was tested for sensitivity by the forward selection procedure (e.g. including and excluding specific variables) with robust standard errors.

**Ethical considerations**: our study was reviewed and approved by the College of Medicine Research and Ethics Committee (COMREC) certificate number (P.07/20/3085). We obtained approval letters for conducting the study in the respective sites from the Hospital Directors. Anonymous identifiers were used to replace actual patient names and their identification with the aim of maintaining privacy and confidentiality.

## Results

**Sociodemographic characteristics of the study participants**: a total of 398 participants were included in the analysis. Table 1, below shows distribution of sociodemographic characteristics and the majority were females, 255 (64%) and married, 251 (67%). Highest proportions of them were in the middle age group (45-54, N=136,34%). The largest category had primary school as the highest attained education, 183 (46%) and were unemployed, 180 (45%). The majority of the participants had neither smoked, 339 (85%) nor taken alcohol, 313 (79%) in their lifetime.

**Table 1:** sociodemographic characteristics by facility (n=398)

Characteristics	Unit of measurement /category	QECH: N=205, n (%)	KCH: N=193, n (%)	Total: N=398, n (%)
**Sex: N (%)**	Female	127 (61.95)	128(66.32)	255 (64.07)
	Male	78 (38.65)	65 (33.68)	143 (35.93)
**Age (years)**		43 ± 12.46	47 ± 12.90	45 ± 12.77
	18-24	15 (7.32)	5(2.59)	20(5.03)
	25-34	29 (14.15)	28(14.51)	57(14.32)
	35-44	69 (33.66)	53 (27.46)	122 (30.65)
	45-54	68 (33.17)	68 (35.23)	136 (34.17)
	55-64	24 (11.71)	39 (20.21)	63 (15.83)
**Marital status**	Never married	15 (7.32)	14 (7.25)	29 (7.29)
	Currently married	137 (66.83)	126 (65.28)	263 (66.08)
	Divorced	31 (15.12)	28 (14.51)	59 (14.82)
	Widow	22 (10.73)	25 (12.95)	47 (11.81)
**Highest formal education level**	No education	35 (17.07)	39 (20.31)	74 (18.64)
	Primary	93 (45.37)	90 (46.88)	183 (46.10)
	Secondary	64 (31.22)	52 (27.08)	116 (29.22)
	Tertiary	13 (6.34)	11 (5.73)	24 (6.05)
**Occupation**	Not employed	102 (49.76)	78 (40.63)	180 (45.34)
	Formally employed	23 (11.22)	17 (8.85)	40 (10.08)
	Informally employed	52 (25.37)	79 (41.15)	131 (33.00)
	Student	5 (2.44)	4 (2.08)	9 (2.27)
	Retired	5 (2.44)	7 (3.65)	12 (3.02)
	Others	18 (8.78)	7 (3.65)	25 (6.30)
**Residential area**	Urban	92 (44.88)	62 (32.29)	154 (38.79)
	Rural	113 (55.12)	130 (67.71)	243 (61.21)
**Socioeconomic status**	Highest	59 (28.78)	37 (19.27)	96 (24.18)
	Higher	35 (17.07)	34 (17.71)	69 (17.38)
	High	27 (13.17)	51 (26.56)	78 (19.68)
	Middle	65 (31.71)	55 (28.65)	120 (30.23)
	Low	19 (9.27)	15 (7.81)	34 (8.56)
**Smoking history**	Never smoked	121 (83.05)	168 (87.05)	339 (85.18)
	Ever smoked	29 (14.15)	22 (11.40)	51 (12.81)
	Current smokers	5 (2.44)	3 (1.55)	8 (2.01)
**Alcohol history**	Never alcohol	156 (76.10)	157 (81.35)	313 (78.64)
	Ever alcohol	43 (20.98)	31 (16.06)	74 (18.59)
	Current alcohol	6 (2.93)	5 (2.59)	11 (2.76)
**Body mass index**	Underweight	32 (15.61)	23 (11.92)	55 (13.82)
	Normal weight	98 (47.80)	128 (66.32)	226 (56.78)
	Over weight	47 (22.93)	32 (16.58)	79 (19.85)
	Obesity	28 (13.66)	10 (5.18)	38 (9.55)

**Common cancers and chronic disease comorbidity prevalence**: Table 2 shows common cancers as follows; cervical, 121 ( 30%), Kaposi´s sarcoma, 97 (24%) followed by breast cancer, 42 (11%), esophageal, 17 (4%) and leukemia, 16 (4%), non-Hodgkin´s lymphoma, 11 (2.76). Most cancer patients had chronic disease comorbidities 245 (61.56%).

**Table 2:** summary of common cancers and comorbidities by facility (n=398)

Comorbidity count	QECH, N=205			KCH, N=193			Total (%)
	**137 (66.83)**			**108 (55.96)**			**245 (61.56)**
**Cancer**	**No comorbidity, n (%)**	**Comorbidity (%)**	**Total (%)**	**No comorbidity**	**comorbidity**	Total	
Cervical	11 (16.18)	41 (29.93)	52 (25.37)	29 (34.12)	40 (37.04)	69 (35.75)	121 (30.40)
Kaposi´ sarcoma	2 (2.94)	44 (32.12)	46 (22.44)	15 (17.65)	36 (33.33)	51 (26.42)	97 (24.37)
Breast	10 (14.71)	9 (6.57)	19 (9.27)	14 (16.47)	9 (8.33)	23 (11.92)	42 (10.55)
Esophageal	6 (8.82)	6 (4.38)	12 (5.85)	3 (3.53)	2 (1.85)	5 (2.59)	17 (4.27)
Leukemia	4 (5.88)	11 (8.03)	15 (7.32)	1 (1.18)	-	1 (0.52)	16 (4.02)
Non-Hodgkin´ lymphoma	2 (2.94)	5 (3.65)	7 (3.41)	2 (2.35)	2 (1.85)	4 (2.07)	11 (2.76)

**Distribution of chronic disease comorbidities by facility**: Table 3 shows distribution of chronic disease comorbidities among study participants as follows; HIV and AIDS, 173 (43%), depression, 35 (9%) hypertension, 33 (8%), anaemia, 34 (9%), tuberculosis, 25 (6.28), dementia 8 (2.01), Rheumatic disease 11 (2.76) and asthma 8 (2.01).

**Table 3:** distribution of chronic disease comorbidities by facility (n=398)

Condition	QECH: N=205, n (%)	KCH: N=193, n (%)	Total: N=398, n (%)
HIV and AIDS	113 (55.12)	60 (3209)	173 (43.47)
Diabetes	1 (0.49)	1 (0.52)	2 (0.50)
Hypertension	19 (9.27)	14 (7.25)	33 (8.29)
Heart failure	1 (0.49)	1 (0.52)	2 (0.50)
Stroke	1 (0.49)	1 (0.52)	2 (0.50)
Renal failure	1 (0.49)	-	1 (0.25)
Hepatitis	1 (0.49)	-	1 (0.25)
Peptic ulcer disease	-	6 (3.11)	6 (1.51)
Dementia	1 (0.49)	7 (3.63))	8 (2.01)
Rheumatic disease	3 (1.46)	8 (4.15)	11 (2.76)
Depression	3 (1.46)	32 (16.58)	35 (8.79)
Chronic bronchitis	1 (0.49)	-	1 (0.25)
Asthma	3 (1.46)	5 (2.59)	8 (2.01)
Malnutrition	1 (0.49)	4 (2.07)	5 (1.26)
Tuberculosis	18 (8.78)	7 (3.63)	25 (6.28)
Anemia	16(7.80)	18 (9.33)	34 (8.54)
Skin ulcer	3 (1.46)	1 (0.52)	4 (1.01)

**Factors associated with disease comorbidities among cancer patients**: Table 4 below shows the distribution of some patients´ characteristics and their association with likelihood of having a disease comorbidity.

**Table 4:** factors associated with disease comorbidity among cancer patients (n=398)

Factor	Unadjusted OR (95% CI)	P-value	Adjusted OR (95% CI)	P-value
**Sex**				
Female	1.00 (ref)*		1.00 (ref)*	
Male	1.10 (0.72-1.67)	0.672	0.70 (0.33-1.47)	0.347
**Age**				
18-24	1.00 (ref) *		1.00 (ref) *	
25-34	1.21 (0.43-3.38)	0.717	0.82 (0.19-ï¿½3.55)	0.794
35-44	1.50 (0.58-3.91)	0.403	1.25 (0.31-ï¿½4.99)	0.751
45-54	1.55 (0.60-4.00)	0.366	1.73 (0.42-ï¿½7.14)	0.449
55-64	0.84 (0.31-2.32)	0.743	0.74(0.11-3.28)	0.690
**Socioeconomic status**				
Highest	1.00 (ref)*		1.00 (ref)*	
Higher	1.41 (0.72-2.74)	0.316	1.91 (0.83-4.43)	0.130
High	0.83 (0.45-1.52)	0.538	1.09 (0.472.51)	0.845
Middle	0.86 (0.49-1.50)	0.595	1.24 (0.55-2.82)	0.608
Low	0.57 (0.26ï¿½1.26)	0.168	1.05 (0.35 -3.14)	0.934
**Education**				
No education	1.00(ref) *		1.00 (ref) *	
Secondary	1.37 (0.75 -2.50)	0.304	1.18 (0.51-2.75)	0.701
**Occupation**				
No employment	1:00 (ref)*		1.00 (ref)*	
Formal employment	2.69 (1.21-5.99)	0.015	4.36 (1.51-11.57)	0.006
**BMI**				
underweight	1:00 (ref)*		1.00 (ref)*	
obesity	2.05 (0.85-4.93)	0.111	3.80 (1.23-11.73)	0.020
**Alcohol history**				
Never	1.00 (ref)*		1.00 (ref)*	
Ever alcohol	1.40 (0.82-2.4)	0.215	1.17 (0.46-2.96)	0.740
Current alcohol	1.79 (0.47-6.90)	0.393	2.25 (0.37-13.72)	0.380
**Smoking history**				
Never	1.00 (ref)*		1.00 (ref)*	
Ever smoke	1.29 (0.69-2.40)	0.420	1.56 (0.60-4.04)	0.362
Current smoker	1.08 (0.25 -4.58)	0.921	1.91 (0.20-17.98)	0.572
**Cancer diagnosis**				
Kaposi sarcoma	1.00 (ref)*		1.00 (ref)*	
Cervical	0.43 (0.23-0.82)	0.011	0.40 (0.17-0.92)	0.032
Esophageal	0.19 (0.06-0.56)	0.003	0.17 (0.05-0.58)	0.005
Bladder	0.16 (0.07-0.72)	0.025	0.40 (0.03-0.54)	0.015
Breast	0.16 (0.07-0.36)	0.025	0.09 (0.03-0.25)	0.000

## Discussion

Chronic disease comorbidities are common among cancer patients in most parts of the world but data in Malawi were limited. This study has found higher proportions of chronic disease comorbidities (62%) among cancer patients (Table 3). The mean age of the participants was 45 years (SD±12.77) meaning most of cancer patients attending to these facilities were in middle age group and it was perhaps due to shorter life expectancy at birth estimated at 63.8 years [14]. Most patients in the study had no history of smoking and the findings mirrored low national prevalence levels of smoking which was 11.2% among adults population studied [21]. There was equally low prevalence of alcohol consumption resembling low estimate for the country what was at17% among adult population [21].

The pattern of common cancers in the study was similar to previous studies conducted in Malawi [6,9]. The study had found that cervical cancer was the commonest cancer in these facilities; Malawi has among the highest incidence rates of cervical cancer across the globe [6]. Although most chronic diseases were investigated in this group of patients, only four conditions had prevalence estimates above 5% (Table 3). However, chronic disease data was collected mainly through self-reports, patient files and health passports, which had potential for underdiagnosis due to limited human and diagnostic capacities in our health facilities. We noted higher prevalence of HIV and AIDS among cancer patients than in general population partly because the two common cancers (cervical and Kaposi´s sarcoma) in this study were AIDS defining malignancies. Further reports indicated that prevalence of hypertension in this study was relatively lower than in general population (8% versus 16%) according to recent unpublished STEPS survey report in 2017 [21]. This could be due to selection bias as healthier cancer patients are likely to survive and therefore attend healthcare. Sicker patients may already be deceased.

We also observed that prevalence of depression in this study was lower than previous national estimate which was between 19% and 30% among adult primary healthcare attendees in Malawi [22,23]. This could be due to underdiagnosis due to inadequate diagnostic knowledge among health workers as a result of poorly integrated mental health care in cancer clinics Malawi [24]. The study had found two factors were associated with higher likelihood of having disease comorbidities and these included: formal employment (p<0.01) and obesity (p<0.02). These results were similar to other studies in Sub-Saharan region [9,15]. Interestingly, some cancers namely; cervical (p<0.03), esophageal (0.01), breast (p<0.01) were protective against having comorbidities. This could be due to survival bias, i.e. sicker patients and individuals with these cancers may not survive longer. Although the study was dominated by women patients, there were no differences in chronic disease prevalence estimates between males and females (Table 4). Similarly, there was no association between age and disease comorbidity which was in sharp contrast to reports from other countries which reported that advanced age was significantly associated with disease comorbidities among cancer patients [7,9,17]. However, our findings did not underrate the importance of ageing in influencing association between cancer and comorbidities but rather could be attributed to a combination of young population of cancer patients and short lifespan in Malawi [14]. Usually, the influence of ageing and development of cancers and other disease comorbidities are more pronounced above the age of 65 years [7,9].

**Limitations**: the study was limited by inability to use comorbidity Index Score because at the time of the study, none was validated in Malawi. The study depended on self-reports for chronic disease comorbidities which were liable to underdiagnoses of these conditions. However, the study has provided meaningful findings as a basis for future studies among cancer patients in Malawi.

## Conclusion

Chronic disease comorbidities were highly prevalent among cancer patients in Malawi. Formal employment and obesity were significantly associated with increased odds of having other chronic diseases among cancer patients. There was need to approach cancer patients with a multidisciplinary focus bearing in mind of other undiagnosed chronic conditions that needed attention for their improved health and outcomes.

### What is known about this topic

- Cancer patients suffer several other chronic diseases;
- Advanced age, positive alcohol and smoking history, obesity was among the factors associated with increased likelihood of having chronic disease comorbidity among cancer patients in other parts of the world.

### What this study adds

- High prevalence of comorbidities (62%) among cancer patients in Malawi;
- Formal employment and obesity were associated with increased odd of having comorbidities among cancer patients in Malawi.
